# Phase-Transformation-Induced Extra Thermal Expansion Behavior of (Sr_*x*_Ba_1–*x*_)TiO_3_/Cu Composite

**DOI:** 10.1038/srep27118

**Published:** 2016-06-03

**Authors:** Jie Sheng, Lidong Wang, Shouwei Li, Benke Yin, Xiangli Liu, Wei-Dong Fei

**Affiliations:** 1 State Key Laboratory for Advanced Welding and Joining, Harbin Institute of Technology, Harbin 150001 P.R. China; 2 Department of Materials Science and Engineering, Shenzhen Graduated School, Harbin Institute of Technology, Shenzhen 518055, P.R. China

## Abstract

The properties of metal matrix composites (MMCs) can be optimized effectively through adjusting the type or the volume fraction of reinforcement. Generally, the coefficient of thermal expansion (CTE) of MMCs can be reduced by increasing the volume fraction of the reinforcement with lower CTE than metal matrix. However, it is great challenge to fabricate low CTE MMCs with low reinforcement volume fraction because of the limitation of reinforcement CTEs. Sr_*x*_Ba_1−*x*_TiO_3_ (SBT) powder presents negative thermal expansion behavior during the phase transformation from tetragonal to cubic phase. Here, we demonstrate that the phase transformation of SBT can be utilized to reduce and design the thermal expansion properties of SBT particle-reinforced Cu (SBT/Cu) composite, and ultralow CTE can be obtained in SBT/Cu composite. The X-ray diffraction analysis on heating indicates that the temperature range of phase transformation is extended greatly, therefore, the low CTE can be achieved within wide temperature range. Landau-Devonshire theory study on the phase transformation behaviors of SBT particles in the composite indicates that thermal mismatch stress significantly affects the Curie temperature of SBT particles and the CTE of the composite. The results given in the present study provide a new approach to design the MMCs with low CTE.

Metal matrix composites (MMCs) have been intensively studied in the past few decades because of their excellent adjustable thermalphysics properties, such as a low coefficient of thermal expansion (CTE) and high thermal conductivity (TC)^1^,^2^. Copper, as a class of important metal matrix, simultaneously exhibits a high TC (up tp 400 W/(m·K)) and CTE^3^,^4^,^5^, conversely, ceramic reinforcement always enhibits a low TC and CTE. However, in order to meet the low CTE demand in application, MMCs generally have high volume fraction of reinforcement (general higher than 50 Vol. %), which inevitability induces the deterioration of thermal conductivities, ductility and machinable properties of MMCs. Therefore, the bottleneck for MMC applications in the fields of electronic packaging, heat sinking, etc is how to achieve a low CTE at the condition of low volume fraction for the optimization of thermal expansion and thermal conductivities^6^,^7^,^8^.

As mentioned before, ceramic-reinforced copper matrix composites have been investigated and developed, such as SiC/Cu. The volume fraction of SiC reinforcement in the SiC/Cu composite is generally very high in order to obtain a sufficiently low CTE, but the TC of the composite is degraded^9^,^10^. It is very difficult to effectively enhance the TC of a SiC/Cu composite at such a high volume fraction of SiC reinforcement. Diamond particles with a low CTE and high TC is also a kind of significant reinforcements which could simultaneously achieve the low thermal expansion and high TC in Cu matrix composites^11^,^12^,^13^. However, superficial oxidation (>700 °C) and graphitization (>1000 °C) have to be considered during the manufacturing process with high sintering temperature^7^.

Generally, it is convenient method to utilize ceramic reinforcement with a low or negative CTE to reduce the ceramic volume fraction in Ceramic/Cu composite, and then a low CTE and high TC can be simultaneously obtained for the composite. In the last decade, many studies have investigated negative-CTE ceramics to reinforce MMCs with a low CTE and high TC, such as β-eucryptite (AlLiSiO_4_)^14^,^15^,^16^,^17^ and ZrW_2_O_8_^18^,^19^,^20^. However, the CTE of β-eucryptite (approximately −0.7 × 10^−6^ K^−1^) is not as low as expected and microcracks are easily formed in the bulk β-eucryptite ceramics due to its poor strength. It is difficult to obtain a good combination of properties in β-eucryptite/Cu composites. Even though ZrW_2_O_8_ exhibits a very large negative CTE (−9 × 10^−6^ K^−1^), for ZrW_2_O_8_–Cu composite, its CTE was significantly larger than the theroy prediction because of the allotropic transformation of ZrW_2_O_8_ under high mismatch stress in the composite. Furthermore, the complex synthesis technology and thermal instability also limit its practical application as the reinforcement in copper matrix composites^7^. Novel materials or mechanisms are expected to develop low CTE and high TC composites.

Utilization of the negative thermal expansion of phase transformation is another promising method to achieve low-CTE composites. For instance, Invar alloy (Fe–36Ni) presents very low CTE and is widely used in the fields of wireless communication, precision instruments, etc.^21^. However, the application of Invar alloys in emerging fields has been restricted owing to their disadvantages such as a high density, low TC, and high resistance.

Sr_*x*_Ba_1−*x*_TiO_3_ (SBT), as an important ferroelectric ceramic, has been intensively investigated. The CTE of SBT can reach as low as −150 × 10^−6^ K^−1^ when the ferroelectric–paraelectric (tetragonal–cubic) phase transformation takes place^22^,^23^,^24^. On the one hand, the Curie temperature (*T*_C_) of SBT is in the range of approximately 20 to 400 K, which can be precisely controlled by adjusting the Sr content^22^, suggesting that we can obtain large negative CTE in a temperature range near room temperature by using SBT powders with different Sr contents. Consequently, the dependence of the CTE of the SBT/Cu composite on the temperature can be designed by the Sr content. On the other hand, the Curie temperature of SBT is very sensitive to the stress^23^, and a thermal mismatch stress is an intrinsic feature of MMCs^25^. Therefore, a wide temperature range is expected for phase transformation owing to the non-uniform stress acting on the SBT particles with different sizes and shapes in the SBT/Cu composite. Based on the performance of SBT during the process of transformation, SBT may act as a promising and newly-developing kind of reinforcement for low CTE and high TC MMCs used at near room temperature.

In the present study, the thermal expansion behavior of an SBT/Cu composite with an SBT volume fraction of 40% has been investigated, and a very low CTE was achieved. The results presented in the paper provide a route to design MMCs with a low CTE. Sr_*x*_Ba_1−*x*_TiO_3_ powders with *x* = 0.1, 0.2, and 0.3 are denoted as 10SBT, 20SBT, and 30SBT, and the corresponding composites are defined as 10SBT/Cu, 20SBT/Cu and 30SBT/Cu composites, respectively.

## Results and Discussion

**Figure 1:**
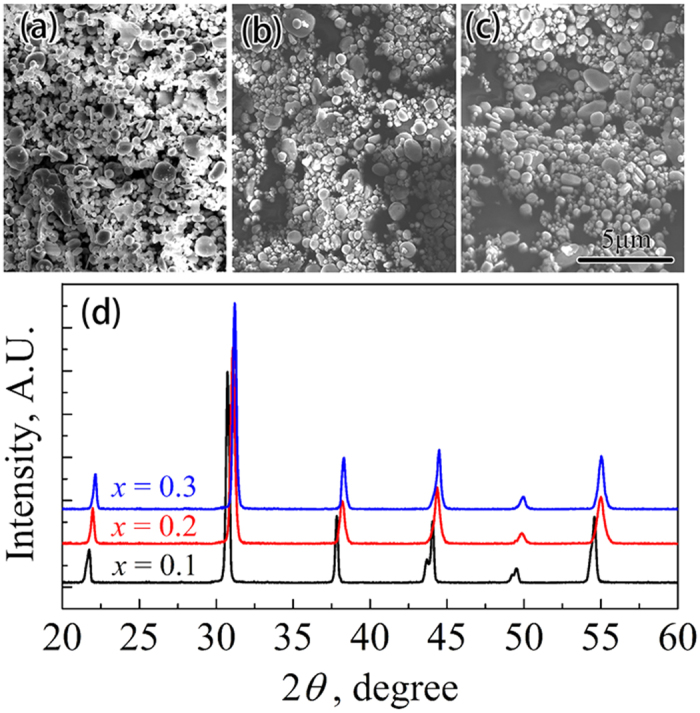
(**a–c**) SEM images of SBT powders with different Sr contents. (**d**) XRD patterns of SBT powders with different Sr contents.

Figure 1(a–c) show the morphologies of SBT powders with different Sr contents observed by scanning electron microscopy(SEM). It is found that the morphologies of the SBT powder are very similar, and the particle size of the SBT powder is very inhomogeneous and distributed from several hundred nanometers to several micrometers. Figure 1(d) presents the X-ray diffraction(XRD) spectra of SBT powders with different Sr contents. Only diffraction peaks corresponding to the perovskite structure can be observed in Fig. 1(d), which indicates that the powders obtained through the above solid reaction method are of pure perovskite phase.

**Figure 2:**
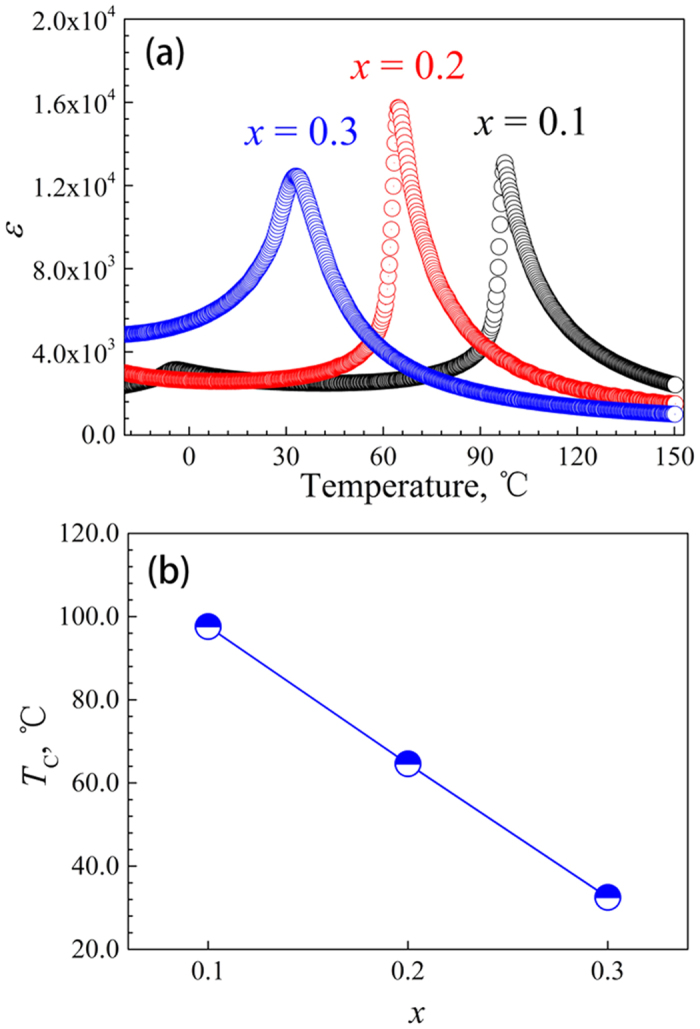
(**a**) Dielectric-constant–temperature curves of SBT powders with different Sr contents. (**b**) Curie temperature of SBT powders with different Sr contents.

In order to determine the values of Curie temperature(*T*_C_) of the SBT powders with different Sr contents, the dielectric-constant–temperature (ε–T) curves were measured and are shown in Fig. 2(a). *T*_C_ can be determined by the peak of ε–T curves. As shown in Fig. 2(b), the *T*_C_ gradually decreases for the SBT powders as the Sr content increases, which is in agreement with previous studies^26^.

**Figure 3:**
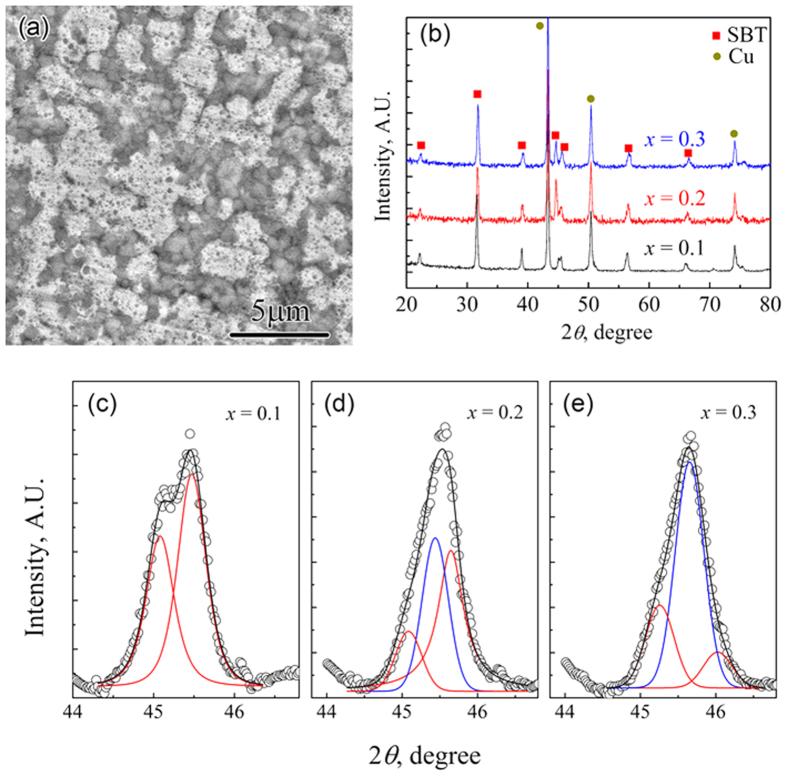
(**a**) SEM image of an SBT/Cu composites. (**b**) XRD patterns of SBT/Cu composites with different Sr contents. (**c–e**) Fine-scan XRD curves in the 2*θ* range of 44–47° of SBT/Cu composites with different Sr contents.

Figure 3(a) shows the typical morphology of SBT/Cu composites with different Sr contents. The SBT powders are uniformly distributed in the composites; moreover, no pores can be observed in the composites. Figure 3(b) shows the XRD spectra of the SBT/Cu composite with different Sr contents, in which only Cu and SBT diffraction peaks are observed. The XRD spectra indicate that no interfacial reaction and other impurities were introduced during the spark plasma sintering(SPS) process.

Fine scan XRD curves in the 2*θ* range of 44–47° were measured at 25 °C to probe the phase composition of the SBT particles in the composites. A single diffraction peak of {200}_C_ exists for cubic(C)–phase SBT exists in this 2*θ* range, and the two diffraction peaks of {002}_T_ and {200}_T_ exist for tetragonal(T)–phase SBT in this 2*θ* range (the subscripts C and T indicate the C and T phases, respectively). Therefore, the phase composition of the SBT powders in the composites can be determined by peak fitting. After *Kα*_*2*_ diffraction striping, the diffraction peaks were fitted by the Pearson VII function. As shown in Fig. 3(c–e), three diffraction peaks are observed for the SBT particles in the composite in this 2*θ* range, which indicates that the C and T phases of the SBT ferroelectric coexist. Among the three peaks, the bilateral peaks arise from the T-phase particles, and the middle peak represents the C phase. The SBT powders in the composites consist of T and C phases, which are different from the as-sintered SBT powders. The Curie temperature of the as-sintered SBT powder in the 20SBT/Cu composite is approximately 65 °C, as shown in Fig. 2(b). However, C-phase SBT particles can be observed at 25 °C in the composite. This suggests that the Curie temperatures of the SBT particles are decreased owing to the thermal mismatch stress in the composite^27^. In addition, the shapes and sizes of the SBT powders and the interfacial state between the SBT powder and the copper matrix are inhomogeneous. Therefore, the mismatch stress acting on different SBT particles in the composites may be quite different, resulting in different Curie temperatures for SBT particles with a different stress, which causes the coexistence of the T and C phases.

**Figure 4:**
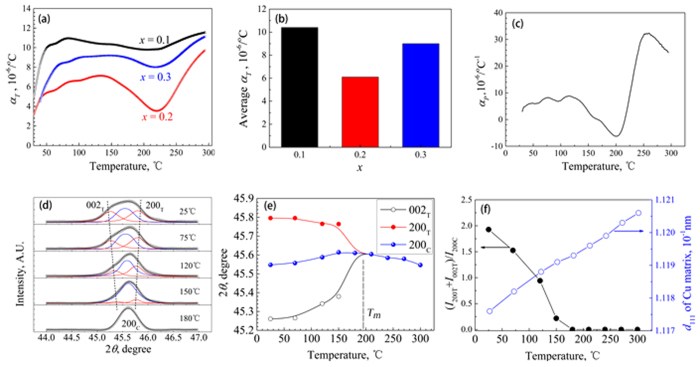
(**a**) Technical CTEs of SBT/Cu composites with different Sr contents. (**b**) Average technical CTEs of SBT/Cu composites with different Sr contents. (**c**) Physical CTE of the 20SBT/Cu composite. (**d**) XRD peaks of 20SBT/Cu composite and their peak fittings at different temperatures. (**e**) Variation in the XRD peak positions of the 20SBT/Cu composite for 2*θ* = 44–47°. (**f**) *I*_T_/*I*_C_ and *d*_111_ of the Cu versus temperature.

Figure 4(a,b) show the technical and average CTEs in the temperature range of 50–300 °C for SBT/Cu composites with different Sr contents. Generally, the thermal expansion behavior can be described by the technical CTE (*α*_*T*_) and physical CTE (*α*_*P*_): with 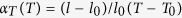 and 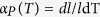, respectively, where *l*_0_ is the sample length at the reference temperature (*T*_0_), and *l* is the sample length at the temperature *T. α*_*T*_ reflects the total expansion rate in the temperature range of *T*–*T*_0_, and *α*_*P*_ is the instantaneous expansion coefficient at *T*. It can be found that the SBT/Cu composites exhibit a very low CTE, and the CTE of the 20SBT/Cu composite is much lower than those of the other two. The above results indicate that there is a suitable Sr content that obtains the optimized thermal expansion properties of the SBT/Cu composites. In addition, the CTE curves rapidly decrease from approximately 150 °C, and form a valley within the temperature range of 150–275 °C. The physical CTE curve of the 20SBT/Cu composite is shown in Fig. 4(c). It is found that the physical CTE is negative in the temperature range where the technical CTE curve rapidly decreases in Fig. 4(a). The thermal expansion behavior of the SBT/Cu composite is quite different from conventional MMCs such as MMCs reinforced by SiC and Al_2_O_3_ particles. In paricular, a negative CTE was rarely observed in MMCs.

In order to study the origin of the low CTE, an *in-situ* XRD analysis of the 20SBT/Cu composite upon heating was carried out in the 2*θ* range from of 44–47°, and the typical XRD curves (after *K*α_2_ diffraction striping) and their peak fittings are presented in Fig. 4(d). According to the XRD peak fittings, the positions of the {200}_C_, {002}_T_, and {200}_T_ peaks of the 20SBT particles in the composite can be obtained, and the lattice constants of the C and T phases can be evaluated. The value of lattice constant for a-axis(*a*_T_) increases, at the same time, the value of lattice constant for c-axis(*c*_T_) decreases as the sample is heated, as shown in Fig. 4(e). The negative expansion of *c*_T_ leads to a low thermal expansion of the SBT particles and composites. *a*_T_ and *c*_T_ are equal to *a*_C_ when the temperature is higher than *T*_*m*_, as shown in Fig. 4(e), which means that the all of the 20SBT particles transform to the C phase. Because *T*_*m*_ is higher than *T*_*C*_ of the 20SBT powders, the Curie temperatures of some 20SBT particles in the composite increase upon heating. This is different from the behavior upon cooling and will be analyzed later in this paper.

The intensity ratio (*I*_T_/*I*_C_) between the T-phase diffraction peaks (the sum of the {200}_T_ and {002}_T_ peak intensities) and the C-phase {200} diffraction peak reflects the content ratio between the T and C phases in the 20SBT/Cu composite. The relationship between *I*_T_/*I*_C_ and the temperature upon heating is presented in Fig. 4(f). It is found that the content of the T-phase 20SBT in the composite decreases as the temperature increases, which indicates that a phase transformation occurs, starting from room temperature to *T*_*m*_. When comparing Fig. 4(c) with Fig. 4(e), the variation trend in the thermal expansion matches the process of SBT phase transformation in the composite. The CTE of the SBT powder is lower than that of Cu matrix; moreover, the CTE of the SBT powder is sufficiently low to negative value when the ferroelectric–paraelectric phase transformation happens. Therefore, the SBT/Cu composites exhibit very low CTEs.

On the basis of previous studies^25^,^28^, the CTE of an MMC can be expressed by the following equation:


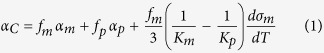


where *α*_*p*_ is the average CTE of the SBT powder; *α*_*m*_ is the CTE of the Cu matrix; *f*_*m*_ and *f*_*p*_ represent the volume fractions of the matrix and particles, respectively; *K*_*m*_ and *K*_*p*_ represent the bulk moduli of the matrix and particle reinforcement, respectively; and *σ*_*m*_ is the thermal mismatch stress in the Cu matrix. It is clear that the CTE of the composite is affected by the CTE and volume fraction of the powder and the rate of change in the matrix. When the SBT particles in the composite transform from the T phase to the C phase, the average CTE of the SBT is dramatically reduced, which leads to a decrease in the CTE of the composite.

In addition, the compressive stress and the tensile stress are simultaneously induced on the SBT particles and on the Cu matrix, respectively, when the composite is cooled from a high temperature because the CTE of the SBT particles is lower than that of the Cu matrix^14^,^15^. The tensile stress acting on the matrix can be relaxed upon heating, and *K*_*m*_ is much smaller than *K*_*p*_ in the SBT/Cu composite; therefore, it can be concluded that the CTE of the composite decreases during this process on the basis of Equation (1) and our previous studies^16^,^17^,^25^. The SBT phase transformation from the T phase to the C phase leads to a sharp decrease of SBT particle CTE; correspondingly, the quick relaxation of the tensile stress causes the CTE of the Cu matrix to decrease greatly, as shown in Fig. 4(f). Therefore, a negative physical CTE can be obtained in the temperature range in which many SBT particles quickly transform from the T phase to the C phase. Moreover, the phase transformation of the SBT powders in the composite occurs over a wide temperature range owing to the inhomogeneous stress in different SBT particles, resulting in a low CTE for the composites over a wide temperature range.

On the basis of the above results, it is found that the abnormal thermal expansion behavior of the SBT/Cu composites is closed to the phase transformation. The most important factor affecting the Curie temperature of the SBT particles in the composite is the thermal mismatch stress, and the effect of the stress on *T*_C_ can be analyzed by Landau–Devonshire theory^29^. The XRD analysis results indicate that the effect of the thermal stress on the Curie temperature upon cooling is different from that during heating process. The difference is analyzed in the following paragraphs.

SBT particles in the composite are in the highly symmetric cubic phase when the temperature is higher than the Curie temperature during the cooling process. Thus, it can be assumed that the SBT particles are isotropic inclusions in the SBT/Cu composites. According to the Eshelby approach of equivalent inclusions for composites^29^, a hydrostatic compressive stress can be induced on the SBT particles upon cooling.

In terms of Landau–Devonshire theory, the Gibbs energy of one SBT particle in the composite is expressed by the following equation^30^,^31^,^32^,^33^.


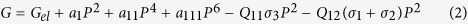


where *P* is the spontaneous polarization along the [001] direction of the particle (*P*_1_ = *P*_2_ = 0, *P*_3_ = *P*); *a*_1_, *a*_11_, and *a*_111_ are given in ref. 28; and *G*_*el*_ is the elastic energy in an SBT particle. It can be considered that the SBT particle experiences a hydrostatic stress (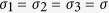). Thus, *P* and *T*_C_ can be determined by as follows:


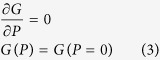


*T*_C_ of an SBT particle in the composite is expressed as


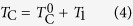


where 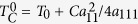, C is the Curie-Weiss constant, T_0_ is the Curie-Weiss temperature, and 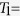

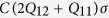
. 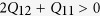 for a pure BT particle according to the data given in ref. 30, assuming that there is no change in this equation when Ba is substituted with Sr atoms, so the *T*_C_ of the SBT particles in the composites decreases upon cooling. Although the *T*_C_ of the as-sintered 20SBT powder is approximately 60 °C, *T*_C_ of the SBT particles in the composite is different owing to the inhomogeneous stress on different SBT particles, which causes the coexistence of the C and T phases at room temperature.

When the temperature is below the Curie temperature, the T-phase SBT particles in the composite are anisotropic, and their stress state can be assumed to be symmetric about the *c* axis, i.e., 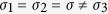. In this case, *T*_C_ of a T-phase SBT particle can be derived from Equations (2) and (3), and


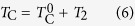


where, 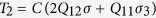. The CTE of T-phase SBT particles in the SBT/Cu composites along [001] direction is negative; thus, the compressive stress acting on the SBT particles along the [001] direction relaxes much quickly, and even transforms into a tensile stress upon heating. Meanwhile, 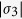 decreases rapidly. When 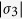 decreases to a certain value, the value of *T*_2_ can be changed to be positive within a certain temperature range; that is, *T*_C_ can be increased upon heating. The Curie temperature is not the same in all SBT particles in the SBT/Cu composites owing to the different particle shape and interface states. Therefore, the T–C phase transformation can affect the CTEs of the SBT/Cu composites over a wide temperature range.

The negative CTE of the 20SBT/Cu composite may be caused by the following two reasons. On the one hand, 20SBT particles exhibit a negative thermal expansion during the phase transformation; then, the transformation of large number of 20SBT particles leads to a quick decrease in the CTEs of the composite. On the other hand, the yield strength of the Cu matrix decreases as the temperature increases, which results in the quick relaxation of the tensile stress on the Cu matrix through plastic deformation^34^. The high rate of change in the stress acting on the Cu matrix causes a further decrease in the CTE of the composite; therefore, a negative CTE is obtained in the 20SBT/Cu composite within a certain temperature range.

In summary, we have designed and prepared SBT-reinforced Cu matrix composites and observed extra low thermal expansion behavior in SBT/Cu composites. Owing to the considerable negative thermal expansion effect during the ferroelectric-paraelectric phase transformation process, the CTE of the SBT/Cu composite can be dramatically reduced and precisely controlled by adjusting the volume fraction of SBT reinforcement. This is the first demonstration of the utilization of this phase transformation to design and reduce the CTE of MMCs, which provides significant application value and an innovative design approach to achieving low-CTE MMCs.

## Methods

### Fabrication of SBT Powders

SBT powders with different Sr contents were prepared through solid reaction method. Chemical pure BaCO_3_, SrCO_3_ and TiO_2_ powders were mixed by ball-milling method for 12 h, and sintered at 1200 °C for 2 h in order to obtain SBT powders.

### Fabrication of SBT/Cu Composite Powders

Pure Cu and SBT powder were mixed through ball-milling for 4 h, with ball-to-powder mass ratio equals 5:1.

### Consolidation of the SBT/Cu Composites Powders

The SBT/Cu composites with the SBT volume fraction of 40% were prepared by spark plasma sintering (SPS) method at 600 °Cand 50 MPa for 10 min, under the condition of vaccum.

### Characterizations

Microstructures of SBT powders and SBT/Cu composites were observed on a Helios Nanolab600i scanning electron microscope (SEM). The phase compositions of SBT powders and composites were analyzed by X-ray diffraction (XRD) on a Philips X’Pert X-ray diffractometer with Cu Kα radiation. The *in situ* XRD on heating was carried out on a PANalytical diffractometer with Cu Kα radiation. Thermal expansion experiments were performed on a Netzsch DIL 402C dilatometer with a heating rate of 2 K/min. The dimension of specimens for thermal expansion measurement is Ф6 mm × 15 mm. The dielectric properties of SBT powders were analyzed on an Agilent 4294A impedance analyzer.

## Additional Information

**How to cite this article**: Sheng, J. *et al.* Phase-Transformation-Induced Extra Thermal Expansion Behavior of (Sr_*x*_Ba_1−*x*_)TiO_3_/Cu Composite. *Sci. Rep.* **6**, 27118; doi: 10.1038/srep27118 (2016).
